# Supplementary material for: HIVprotI: an integrated web based platform for prediction and design of HIV proteins inhibitors
Source: J Cheminform. 2018 Mar 9;10:12. doi: 10.1186/s13321-018-0266-y (PMC5845081; doi:10.1186/s13321-018-0266-y)
Supplement: Supplementary file 2 — Additional file 2. Source code of HIVProtI web server. [file 13321_2018_266_MOESM2_ESM.zip › HIVprotI_Source-code/hivprotI/weka-3-6-6/doc/weka/classifiers/bayes/net/search/local/Scoreable.html]

Scoreable


---


|  |  |  |
| --- | --- | --- |
| **Overview** \| **Package** \| **Class** \| **Tree** \| **Deprecated** \| **Index** \| **Help** \| **Weka's home** | |  |
| **PREV CLASS**   **NEXT CLASS** | **FRAMES**    **NO FRAMES**     **All Classes** |  |
| SUMMARY: NESTED \| FIELD \| CONSTR \| METHOD | DETAIL: FIELD \| CONSTR \| METHOD |  |


---


## weka.classifiers.bayes.net.search.local Interface Scoreable

**All Known Implementing Classes:**: DiscreteEstimatorBayes, DiscreteEstimatorFullBayes

---

``` public interface Scoreable ```

Interface for allowing to score a classifier

**Version:**
:   $Revision: 1.4 $

**Author:**
:   Remco Bouckaert (rrb@xm.co.nz)

---

| **Field Summary** | |
| --- | --- |
| `static int` | `AIC` |
| `static int` | `BAYES`             score types |
| `static int` | `BDeu` |
| `static int` | `ENTROPY` |
| `static int` | `MDL` |


| **Method Summary** | |
| --- | --- |
| `double` | `logScore(int nType, int nCardinality)`             Returns log-score |

**Field Detail**

### BAYES

```
static final int BAYES
```

:   score types

    **See Also:**: Constant Field Values

---


### BDeu

```
static final int BDeu
```

**See Also:**: Constant Field Values

---


### MDL

```
static final int MDL
```

**See Also:**: Constant Field Values

---


### ENTROPY

```
static final int ENTROPY
```

**See Also:**: Constant Field Values

---


### AIC

```
static final int AIC
```

**See Also:**: Constant Field Values


**Method Detail**

### logScore

```
double logScore(int nType,
                int nCardinality)
```

:   Returns log-score

    :   **Parameters:**: `nType` - the score type **Returns:**: the log-score


---


|  |  |  |
| --- | --- | --- |
| **Overview** \| **Package** \| **Class** \| **Tree** \| **Deprecated** \| **Index** \| **Help** \| **Weka's home** | |  |
| **PREV CLASS**   **NEXT CLASS** | **FRAMES**    **NO FRAMES**     **All Classes** |  |
| SUMMARY: NESTED \| FIELD \| CONSTR \| METHOD | DETAIL: FIELD \| CONSTR \| METHOD |  |


---
